# [Retracted] Reduced expression of Snail decreases breast cancer cell motility by downregulating the expression and inhibiting the activity of RhoA GTPase

**DOI:** 10.3892/ol.2025.15143

**Published:** 2025-06-16

**Authors:** Ali Zhang, Quansheng Wang, Zhiqiang Han, Wei Hu, Ling Xi, Qinlei Gao, Shixuan Wang, Jianfeng Zhou, Gang Xu, Li Meng, Gang Chen, Ding Ma

Oncol Lett 6: 339–346, 2013; DOI: 10.3892/ol.2013.1385

Following the publication of the above paper, it was drawn to the Editor's attention by a concerned reader that two pairs of the control β-actin protein bands shown in one of the western blots in Fig. 4E on p. 340 were strikingly similar to each other, such that it appeared as if these data may have been duplicated within the figure itself. Upon investigating this matter independently in the Editorial Office, the Editor of *Oncology Letters* has decided that this paper should be retracted from the Journal on the grounds of a lack of confidence in the presented data. The authors were asked for an explanation to account for these concerns, but the Editorial Office did not receive a reply. The Editor apologizes to the readership for any inconvenience caused.

